# Efforts to reduce the length of stay in a low-intensity ICU: Changes in the ICU brought about by collaboration between Certified Nurse Specialists as head nurses and intensivists

**DOI:** 10.1371/journal.pone.0234879

**Published:** 2020-06-15

**Authors:** Tomohide Fukuda, Hironori Sakurai, Masanori Kashiwagi

**Affiliations:** 1 Faculty of Nursing, Kyoritsu Women’s University, Tokyo, Japan; 2 Department of Anesthesiology, National Hospital Organization Tokyo Medical Center, Tokyo, Japan; 3 Department of Anesthesiology, Tokyo Saiseikai Central Hospital, Tokyo, Japan; Wayne State University, UNITED STATES

## Abstract

Certified Nurse Specialists (CNS) are advanced practice nurses that often play a role in management. This study aims to investigate whether cooperation between CNSs in the position of Intensive Care Unit (ICU) head nurse and intensivists change the length of stay for ICU patients. A single centered retrospective cohort study design was followed. A multivariable regression analysis was performed to determine whether there is a difference in patients’ length of ICU stay for two years before and after CNS as ICU head nurse and an intensivist started collaborating. The patients’ diagnosis, age, gender, scheduled/emergency admission, surgical history, length of ICU stay, usage of ventilator, and details of ICU treatment were collected from the institution’s electronic medical records. During the study period (April 2015 to March 2019), 3,135 patients were admitted to ICU, with 1,471 in the before collaboration group and 1,664 in the after-collaboration group. Collaboration between the CNS as head nurse and intensivists was significantly associated with shorter length of ICU stay (coefficient -0.03 [95% CI, -0.05–0.01], *p* < 0.001, *t*-statistic -3.29). Our main finding illustrates that in low-intensity ICUs, collaboration between CNSs as head nurses and intensivists may reduce patients’ length of ICU stay.

## Introduction

A low-intensity ICU employs a system in which only doctors in each medical department treat patients in the Intensive Care Unit (ICU) and intensivists get involved only at the request of the attending physician or surgeon. [1] In such ICUs, collaboration between the attending physician, intensivists, the ICU nurse, and other actors is vital. Specifically, the Advanced Practice Nurse (APN) plays a significant role in intensive care, improving bedside care, consultation, and ethical coordination. [2–7]

An APN has a master’s or doctoral degree and contributes to the development of bedside care through evidence-based direct care, multi-professional collaboration, and education. Certified Nurse Specialist (CNS) is one of the titles recognized as an APN in Japan. A CNS received education in a graduate school master’s program and performs nursing practice with the background of knowledge in nursing management, nursing education, and nursing research, as well as their specialized field. The system is modeled on the same principal as that of clinical nurse specialists in the United States. [6,8]

Similar to other countries, the CNS in Japan is expected to provide advanced nursing practice and education and provide care and decision support to patients with complex clinical problems as well as multi-professional teams experiencing difficult issues. Approximately 20% of CNSs work as nurse directors, vice nursing directors, and head nurses [9].

In low-intensity ICUs where no intensivists are stationed, it is necessary to combine the expertise of each profession to treat and care for patients and to improve their condition. Therefore, CNSs who specialize in critical care not only support collaboration among different professions and provide advanced care to patients based on their specialized knowledge, but also participate in organizational management as the head nurse of the ICU.

As their chosen career, experienced CNSs sometimes manage wards as head nurses. The duties of a head nurse include organizational development by being responsible for interpersonal relationships between nurses and patient care, work planning, intrinsic motivation, workload, unit size, and leadership to improve the job satisfaction of nurses and quality of the care. [10] Moreover, to support the recovery of as many ICU patients as possible, effective use of ICU beds—a limited resource in hospitals—is essential. This is one of the important missions of doctors and nurses in charge of ICUs.

The CNS as head nurse has a background of expertise in managing both the organization and patient care. In this position as a clinical care specialist, multidisciplinary collaboration and interdisciplinary collaboration for organizational management are combined. In ICUs, treatment and care are especially critical. Effective collaboration between physicians and nurses is needed as the first step in multidisciplinary collaboration to ensure that appropriate knowledge-based and evidence-based care is provided to improve patient outcomes and the quality of treatment and care. Outcomes and goals are set in collaboration between the head nurse, who is responsible for the ward, and the doctor, who is responsible for the treatment, enabling the entire organization to deliver treatment and care in the same direction.

However, no study has examined patient outcomes associated with intensivists’ and CNSs’ collaborative management of an ICU. The aim of this study is to investigate whether CNSs as head nurses can work with intensivists to make changes to patients’ length of stay in low-intensity ICUs.

## Method

### Research design

A single centered retrospective cohort study was conducted at the ICU of a Japanese Hospital during a 4-year period (April 2015 to March 2019).

### Collaboration between CNS and intensivists

The ICU in the target facility has 10 beds and is a general ICU that accepts patients with sudden changes in their health status from the hospital’s other wards; mainly postoperative patients and tertiary emergency patients. As this is a low-intensity ICU, surgeons were concurrently in charge of the ICU until March 2017; however, from April 2017, the anesthesiology department (the department in which intensivists normally practice) became the center of the ICU’s management and started participating in the treatment of ICU patients while concurrently performing surgery.

In an interview with CNS, several intensivists, and surgeons about the changes in management, the following points were clarified after CNS as head nurse and intensivists were included in the management of the ICU.

1. The system of providing medical care in the ICU was changed from a system in which each department provided medical care by itself to a system in which the attending physician consulted with the intensivists when necessary (so-called “elective care consultations”). [11] Prior to the introduction of elective care consultations, the CNS would discuss issues related to the treatment plan and the patient’s background with the primary physician and make prior arrangements to facilitate the intervention of the intensivists.
2. The CNS as ICU head nurse and intensivists now play central roles in providing nursing education through the preparation for changes in ICU management and the preparation and implementation of various protocols. This involves providing advice and support for treatment in each department and arranging consultations, coordination, and conferences concerning long-term patients and cases with ethical problems.
3. In regular morning meetings, the conditions of all the patients in the ICU were outlined/communicated to the intensivists.
4. Considering ICU bed control, the CNS comprehensively examines the severity of the patients’ conditions and the necessity of daily life support in the ICU to select patients to enter or leave the ward, while the intensivists examines the medical necessities to make the final decision.
5. The CNS as head nurse serves as the link between the primary physician and the intensivists, as well as between the ICU nurse and the anesthesiologist.

**Fig 1:**
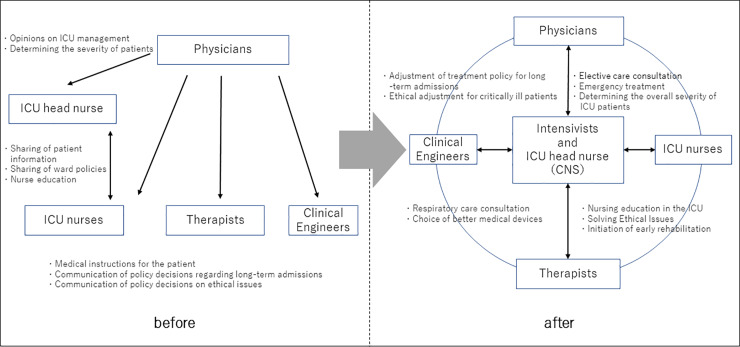
Changes in ICU due to collaboration of CNS as head nurse and intensivists.

As a result of the implementation of these items, the ICU—in which the primary physician used to decide on the treatment plan—has been transformed into a collaborative ICU, in which the CNS and the intensivists determine the treatment plan and the nursing policy of the medical team (Fig 1).

### Data collection

This is a secondary study that uses datasets collected during a previous related study. [12] This means that this study did not involve any intervention; therefore, acquiring informed consent from patients was judged to be unnecessary. Patients admitted to the ICU of the target hospital in Japan between April 2015 and March 2019 are included in the participant group. The patients’ diagnosis, age, gender, scheduled or emergency admission, surgical history, length of ICU stay, severity, and degree of medical and nursing needs were collected from the hospital’s electronic medical records. Patients whose data had missing values were excluded.

Research ethics approval was obtained from the internal review board of Kyoritsu Women’s University (approved ID: KWUIRBA#19011) and the internal review board of the Tokyo Saiseikai Central Hospital (Approved ID: 30–88). Consent was not required from participants due to the de-identified nature of the data and the retrospective study design.

### Measurement

The severity of each patient’s condition and the extent of their required medical and nursing care were determined. The former was assessed using a scale that measures the degree of dependence on medical care for patients admitted to the ICU created by the Japanese Ministry of Health, Labour and Welfare (Supplementary material, S1 and S2 Tables). [12] The higher the score, the greater the degree of dependence on medical treatment and nursing care (severity). The scale is divided into items A and B. Item A scores medical dependency out of a maximum of 15 points. The points are based on the presence or absence of electrocardiogram monitor, infusion pump, syringe pump, A-line, central venous catheter, respirator attachment, use of transfusion and/or blood products, Swan-ganz catheter, and special treatment (intra-aortic balloon pumping; IABP, Continuous hemodiafiltration; CHDF, percutaneous cardiopulmonary support; PCPS, intracranial pressure measurement; ICP measurement, ventricular assist device; VAD, extracorporeal membrane oxygenation; ECMO). Item B scores the degree of dependence on nursing care out of a maximum of 12 points. These points include turning over (unable to, can with assistance, can), transfer (cannot, needs assistance or observation, can), oral care (cannot perform, can perform), food intake (cannot feed self, requires assistance, can feed self), removing clothes (cannot, requires assistance, can), understanding instructions regarding medical treatment (cannot understand, can understand), and dangerous behavior (yes, no). The evaluation criteria for each item apply nationwide and are evaluated daily in patients admitted to the ICU.

ICU nurses were responsible for evaluating patients based on this scale after completing training on how to do so via e-learning. After completing the training, all the nurses passed a computer-based examination. [12]

### Data analysis

The study period was divided into two periods: before collaboration (April 2015 to March 2017) and after collaboration (April 2017 to March 2019) between CNS as head nurse and intensivists. The two periods were compared using a Fisher’s exact test for categorical variables and a Mann-Whitney U test for continuous variables. To determine whether the collaboration between the CNS as head nurse and the intensivists was associated with long-term admission of ICU patients, a multivariable regression analysis was performed. The independent variables were age, gender, and variables that were significantly different in univariate analysis. The dependent variable, the length of ICU stay, was translated logarithmically. This is to avoid affecting the data owing to some long-term ICU admissions. In addition, variables with a Variance Inflation Factor (VIF) value of 10 or more were excluded. Prior to the analysis, the difference in the assignment of ICU nurses between the two periods was examined to consider the possibility of the assignment of ICU nurses affecting the study results. The difference was significant/non-significant with a *p*-value < 0.05. All statistical analyses of the data collected were performed using the Excel statistical software package (BellCurve for Excel; Social Survey Research Information Co., Ltd., Tokyo, Japan).

## Results

### Patient characteristics

Included in the analysis were 3,135 patients admitted to ICU during the study period: 1,471 in the before group and 1,664 in the after group. The patients’ characteristics are summarized in Table 1. The age, sex, and number of patients on a ventilator did not differ between the two groups. The anesthesiology department provided intensive ICU management in the after group and a system was established to allow patients who are at high risk of postoperative complications to admission the ICU for their preoperative medical examination. As a result, the number of postoperative ICU patients increased. There was also an increase in the number of patients with gastrointestinal (*p* < 0.001) and respiratory (*p* < 0.001) diseases. However, the establishment of a new 10-bed ICU in the hospital as a result of ward reorganization led to a decrease in the number of patients with cardiovascular disease (*p* < 0.001). As the number of patients admitted to the hospital after surgery increased, the severity of patients’ medical and nursing needs evaluated by item A of the scale increased (*p* < 0.001), while those evaluated by item B decreased (*p* < 0.001). These scales were weakly correlated in the analysis with Spearman’s rank correlation coefficient (r = .254 *p* < .001). An increase in the number of elective surgery patients also led to a decrease in their ICU length of stay (*p* = 0.001), the number of ventilator users (*p* = 0.001). Furthermore, we aimed to determine whether there was a difference in the number of nurses between the two periods and found that there was no difference between the two groups.

**Table 1:** Patient characteristics.

	before	after	*p*-value
	(*n* = 1,471)	(*n* = 1,664)	
Age (median, range)	71 (14–105)	71 (19–106)	0.30
Gender (Male, %)	1029 (70.0)	1165 (70.0)	0.30
Operation (%)	761 (51.7)	1141 (68.6)	< 0.001
Emergency admission (%)	710 (48.3)	523 (31.4)	< 0.001
Emergency Operation (%)	109 (7.4)	153 (9.2)	0.08
Diagnostic Category (%)			
Cardiovascular	717 (48.7)	596 (35.8)	< 0.001
Gastrointestinal	349 (23.7)	495 (29.7)	< 0.001
Respiratory	120 (8.2)	204 (12.3)	< 0.001
Neurological	58 (3.9)	85 (5.1)	0.12
hematologic	48 (3.3)	59 (3.5)	0.69
others	179 (12.2)	225 (13.5)	0.26
Severity, medical and nursing needs degree for ICU A score (median, range)	4 (1–15)	5 (1–15)	< 0.001
Severity, medical and nursing needs degree for ICU B score (median, range)	8 (2–19)	7 (1–12)	< 0.001
ICU stay (days)	3 (1–60)	2 (1–52)	< 0.001
Patients with mechanical Ventilation (including NPPV, %)	569 (38.7)	544 (33.5)	0.001
Mechanical ventilation days (including NPPV, median, range)	3 (1–46)	3 (1–47)	0.003
ICU nurse staffing (median, range)	25(22–27)	25(20–27)	0.378

Data are presented as median. Fisher’s exact test, Mann-Whitney U test.

NPPV: Noninvasive Positive Pressure Ventilation

### Effects of collaboration between CNS as head nurse and intensivists on long-term admissions in ICU patients

The results of our multivariable regression analysis to determine the length of ICU stay are shown in Table 2. Collaboration between the CNS as head nurse and intensivists was shown to be significantly associated with shorter ICU stay (coefficient -0.03 [95% CI, -0.05–0.01], *p* < 0.001, t-statistic -3.29). Further, there were fewer long-term ICU admissions of males than of females (coefficient -0.03 [95% CI, -0.05 –-0.01], *p* = 0.002, t-statistic -3.10). Emergency admissions (coefficient 0.10 [95% CI, 0.06–.14], *p* < 0.001, t-statistic 5.03), patients on mechanical ventilation (coefficient 0.12 [95% CI, 0.10–0.15], p < 0.001, t-statistic 8.39), and the degree of severity of ICU needs item A score (coefficient 0.03 [95% CI, 0.03–0.04], p < 0.001, t-statistic 12.9) and item B score (coefficient 0.01 [95% CI, 0.01–0.02], p < 0.001, t-statistic 4.76) in patients with cardiovascular, gastrointestinal, and respiratory problems were significantly associated with length of ICU stay.

**Table 2:** Association between CNS and intensivists collaboration and other predictor variables and ICU stay by multivariable regression analysis.

factors	Regression coefficient (95% CI)	*p*-value	*t-*statistic	*VIF*
Collaboration of CNSs and intensivists	-0.03 (-0.05 –-0.01)	< 0.001	-3.29	1.17
Age	0.00(0.00–000)	0.45	0.00	1.03
Gender (0: male)	-0.03 (-0.05 –-0.01)	0.002	-3.10	1.02
Operation (0: absence)	-0.03 (-0.07–0.01)	0.09	-1.69	5.01
Emergency admission (0: absence)	0.10 (0.06–0.14)	< 0.001	5.03	4.92
Patients on mechanical ventilation	0.12(0.10–0.15)	< 0.001	8.39	1.21
Cardiovascular (0: none)	-0.03(-0.06– -0.01)	< 0.001	-2.69	1.93
Gastrointestinal (0: none)	-0.10(-0.12– -0.08)	< 0.001	-7.49	1.98
Respiratory (0: none)	-0.08(-0.11– -0.05)	< 0.001	-5.17	1.50
Severity degree and medical and nursing needs for ICU A score	0.03 (0.03–0.04)	< 0.001	12.9	1.50
Severity degree and medical and nursing needs for ICU B score	0.01 (0.01–0.02)	< 0.001	4.76	1.09
Adjusted R^2^	0.33			

## Discussion

This study aimed to investigate whether collaboration between a CNS as ICU head nurse and intensivists changed the length of ICU stay. The period of collaboration between the CNS as ICU head nurse and intensivists in a single center was shown to be associated with shorter ICU stays for admitted patients.

Prognosis based on knowledge and experience and multidisciplinary treatment and care may not only save the lives of ICU patients with severe and complex conditions, but may also influence their subsequent activities of daily living and quality of life. Intensivists have a high level of expertise in managing the condition of critically ill patients, while CNSs in critical care also performs advanced nursing care, education, and research against the background of their own clinical experience and specialized medical and nursing knowledge [13–15]. This study showed that collaboration between CNSs and intensivists may lead to the establishment of effective treatment styles in a low-intensity ICU.

As a care provider, the CNS as head nurse can clearly explain the nursing perspective to intensivists, surgeons, physicians and other medical personnel and discuss their positions. By predicting the patients’ prognoses and physical/mental function based on the comprehensive judgment of the various personnel members, it is possible to ensure that patients’ treatment and care moves in the best direction from an early stage. Moreover, because CNSs are also APNs, the quality of ICU care will be improved by coordinating treatment policies and nursing care with the physician and intensivists. The CNS as head nurse also sets appropriate unit goals and outcomes while managing and monitoring the unit to improve the quality of care. [16, 17]

As a head nurse, the CNS is able to set more detailed goals and outcomes in managing the organization and make better use of human, physical, and financial resources, thereby attempting to improve care. When the CNS assumes the role of head nurse, they are able to set more detailed and realistic goals and outcomes by utilizing the perspective of an APN. This allows human, physical, and financial resources to be utilized more effectively to enhance patient care.

In a low-intensity ICU, the health care team (consisting of primary doctors, intensivists, ICU nurses, and other medical personnel) needs to have a solid basis and to employ teamwork, with the CNS as head nurse and intensivists managing the organization as the decision makers based on advanced medical and nursing practices. Goals and policies for personnel providing bedside care are realistic and are therefore likely to gain staff acceptance. Moreover, the CNS, in their role as a manager, can seek better policies for each patient by discussing patient conditions and ICU management with the physician, nurse, and other medical personnel from a neutral standpoint, together with intensivists. In addition, by discussing treatment policies among multiple professions, the ICU staff can learn about the interests and perspectives of multiple professions, which is expected to have an educational effect.

This study suggests that intensivists—who were not in charge of patients as primary care physicians—and the CNS as head nurse made a comprehensive change to patients’ conditions and the burden of nursing care. As a result of fair and unbiased treatment and bed control, timely patient flow management in the ICU may be related to reducing the length of ICU patient stay.

A system in which intensivists advise on the treatment of critically ill patients, the CNS manages the ICU as a head nurse, and the patient is treated and involved in complex problems by an APN can be a useful model for low-intensity ICUs. It can contribute to solving patients’ problems through elective care consultation, intervention in teams and personnel facing difficult problems at the appropriate time, advanced care, goal setting, and efficient organizational management.

### Limitations

This study is a retrospective study that utilizes data from one situation; therefore, it may be difficult to apply this result to other institutions. Moreover, the long-term ICU admission of patients is complicated by a number of factors and even though the results point toward collaboration having a positive effect, it cannot be determined whether the collaboration between the CNS as head nurse and intensivists directly affected the long-term ICU admission of patients.

Therefore, it is necessary for future studies to increase the number of target facilities and investigate the details of the collaboration between the CNS and intensivists. There may also be confounding concerning outcomes other than those assumed here. In the future, it will be possible to pursue an ideal ICU while simultaneously dealing with reality by accumulating reports on efforts to improve the quality of medical care and their results in low-intensity ICUs.

## Conclusions

In low-intensity ICUs, collaboration between intensivists and the CNS may change the overall ICU package and reduce the length of ICU stay for patients.

## Supporting information

S1 Table Evaluation sheet for severity, medical care, and nursing necessity for ICU admission (Original version). (XLSX)

S2 Table Evaluation sheet for severity, medical care, and nursing necessity for ICU admission (English version). (XLSX)
